# Analysis of Nanowire pn-Junction with Combined Current–Voltage, Electron-Beam-Induced Current, Cathodoluminescence, and Electron Holography Characterization

**DOI:** 10.3390/mi15010157

**Published:** 2024-01-20

**Authors:** Nicklas Anttu, Elisabetta Maria Fiordaliso, José Cano Garcia, Giuliano Vescovi, David Lindgren

**Affiliations:** 1 Physics, Faculty of Science and Engineering, Åbo Akademi University, FI-20500 Turku, Finland; 2 National Centre for Nano Fabrication and Characterization, Technical University of Denmark, 2800 Kongens Lyngby, Denmark; 3 Sol Voltaics AB, 223 63 Lund, Sweden

**Keywords:** III–V semiconductor nanowire, electron-beam-induced current, cathodoluminescence, current–voltage characterization, electron holography, drift-diffusion modelling

## Abstract

We present the characterization of a pn-junction GaAs nanowire. For the characterization, current–voltage, electron-beam-induced current, cathodoluminescence, and electron holography measurements are used. We show that by combining information from these four methods, in combination with drift-diffusion modelling, we obtain a detailed picture of how the nanowire pn-junction is configured and how the recombination lifetime varies axially in the nanowire. We find (i) a constant doping concentration and 600 ps recombination lifetime in the n segment at the top part of the nanowire; (ii) a 200–300 nm long gradient in the p doping next to the pn-junction; and (iii) a strong gradient in the recombination lifetime on the p side, with 600 ps lifetime at the pn-junction, which drops to 10 ps at the bottom of the p segment closest to the substrate. We recommend such complementary characterization with multiple methods for nanowire-based optoelectronic devices.

## 1. Introduction

III-V semiconductor nanowires [1,2,3,4,5] have received considerable interest for optoelectronic applications such as in solar cells [6,7,8,9,10,11,12], photodetectors [13,14], and light-emitting diodes [15]. In such applications, different regions of the nanowire are intentionally doped with donors and acceptors to induce n- or p-type doping [16,17]. The configuration of the n-type and p-type regions, together with the recombination lifetime of excess charge carriers, affects the optoelectronic response of the nanowire [18,19], as observed for example with current–voltage (IV) measurements on the nanowires. However, in the bottom-up fabrication of nanowires, non-intentional gradients in both doping profile and recombination lifetime can show up [20]. Therefore, assessment of these parameters and their possible spatial variation within the nanowire is of great interest.

Electron-beam-induced current (EBIC) measurements have been used to understand the spatially varying probability for extraction of photogenerated charge carriers in nanowires [20,21,22,23,24,25]. With electron holography, it has been possible to obtain the electrostatic potential gradient inside nanowires [26,27,28]. Cathodoluminescence (CL) measurements can be used for assessing doping concentration and luminescence properties in nanowires [29,30,31].

Here, we perform IV, EBIC, CL, and electron holography measurements on GaAs nanowires that have a pn-junction. We show that by combining information from these four methods, in combination with drift-diffusion modelling, we obtain a detailed picture of how the nanowire pn-junction is configured and how the recombination lifetime varies within the nanowire.

We recommend such complementary characterization with multiple methods for nanowire-based optoelectronic devices. In our opinion, none of the methods by themselves provide sufficient information for efficient development of devices.

## 2. Materials and Methods

### 2.1. Nanowire Growth

**Figure 1:**
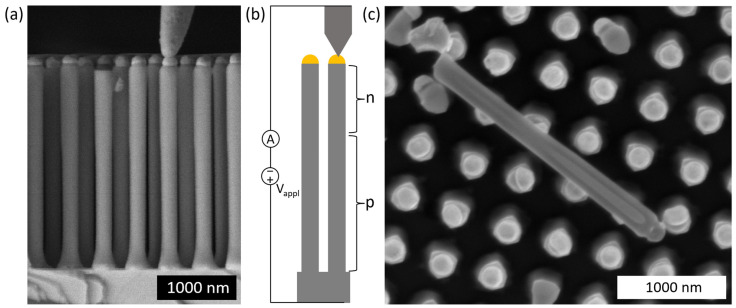
(**a**) SEM image in side-view of as-grown GaAs nanowires with the nanoprobe at the top of a nanowire and the GaAs substrate below the nanowires. (**b**) Schematic of the nanoprobe contacting, which is used in IV, EBIC, and CL measurements. Here, n and p indicate the n and p regions. (**c**) SEM image of a GaAs nanowire left lying flat on the growth substrate when shaving off the nanowires for transfer to TEM grid for electron holography measurements.

The GaAs nanowires are grown using metalorganic vapor-phase epitaxy (MOVPE), in a similar manner as in Ref. [32]. The growth substrate is a p-type GaAs substrate, with approximately 10^16^ cm^−3^ doping concentration, which is patterned with a 500 nm period, square array of Au catalyst particles. In the growth design for the current study, the nanowire growth is intended to switch from p-type dopant to n-type dopant at a height of 1250 nm of a 2500 nm long GaAs nanowire (note that in Ref. [32], in contrast, a deliberate i-region was aimed for, to induce an n-i-p diode configuration). The GaAs core is approximately 160 nm in diameter, and there is an Al*_x_*Ga_1−*x*_As high-bandgap passivation shell layer of approximately *x* = 0.80 and 30–50 nm in thickness. For additional details of the nanowire growth, see Section S1 in the Supplementary Materials. See Figure 1a for a scanning electron microscope (SEM) image of the as-grown nanowires.

### 2.2. IV and EBIC Measurements with Nanoprobe Contacting

We perform IV and EBIC characterization of a single as-grown GaAs nanowire using a nanoprobe (PS4 Prober Shuttle, Kleindiek Nanotechnik, Reutlingen, Germany) inside an SEM (SU8010, Hitachi High-Tech Corporation, Tokyo, Japan) to contact the Au particle at the top of the nanowire (see Figure 1b for a schematic). The same combination of SEM and nanoprobe is used as in Ref. [32]—see Ref. [20] for an example of similar nanoprobe characterization of InP nanowires. The substrate is used as the second contact. For the IV curves, external bias *V*_appl_ is applied and the current is measured. For EBIC measurements, the external bias is kept at *V*_appl_ = 0 V, the electron beam in the SEM is scanned in a point-like manner over the nanowire, and for each measurement point, the current in the external circuit is recorded.

### 2.3. CL Characterization

The CL characterization is performed with a hyperspectral CL imaging system (SPARC, Delmic, Delft, The Netherland) installed to the same SEM as that for IV and EBIC measurements in Section 2.2 above (see Ref. [29] for details of the setup). For this study, the CL measurements are conducted on a single as-grown nanowire, after contacting the nanowire with the above nanoprobe and keeping the external circuit in short-circuit condition (that is, at *V*_appl_ = 0 externally). With the short-circuit condition, we minimize the contribution to CL from excess charge carriers that, after diffusing to the pn-junction, bias the pn-junction, leading to luminescence from the junction region. For the CL spectrum at the middle of the p side and n side, respectively, see Figure S1 in the Supplementary Materials.

### 2.4. Electron Holography Characterization

Off-axis electron holography is a powerful method for dopant assessment in nanostructures [26]. Electron holography is a transmission electron microscopy (TEM) technique that measures a spatially resolved phase difference, Δ*φ*, using interference between electrons that pass through the specimen (object wave) and electrons that pass through the vacuum (reference wave). Δ*φ* is related to the crystal potential, or mean inner potential (MIP) *V*(*x*, *y*, *z*), according to [26]

$$∆\varphi =CE\;\int_{0}^{t}V\left(x,y,z\right)\;dz$$

where *CE* is a microscope acceleration voltage-dependent constant, and *t* is the specimen thickness. *CE* is equal to 8.64 × 10^6^ rad/Vm for a TEM operating at 120 kV.

Here, nanowires are transferred from their substrate to a TEM grid after scratching the surface gently with a tweezer. Holograms are recorded using an FEI Titan 80-300ST field emission gun TEM (ThermoFischer, Waltham, Washington, DC, USA), operating at 120 kV and equipped with a rotatable Möllenstedt biprism.

### 2.5. Drift-Diffusion Modelling

To support the analysis of the characterization results, we perform drift-diffusion modelling for the electron-hole transport. The numerical solving of the drift-diffusion equations is performed with the Semiconductor Module in Comsol Multiphysics. Due to the axial configuration of the pn-diode, we use a one-dimensional (1D) approximation of the nanowire, such that only the axial position enters our simulations. For details and validity of the 1D model, see Ref. [19].

In the modelling, we assume a temperature of *T* = 300 K, use Fermi-Dirac statistics for the carrier concentrations, and, for simplicity, we do not take into account possible carrier-concentration-induced bandgap narrowing in the simulations. Furthermore, for GaAs, we assume a bandgap of 1.424 eV [33], effective conduction band density of states of 4.7 × 10^17^ cm^−3^ [16], and effective valence band density of states of 9.0 × 10^18^ cm^−3^ [16].

We perform two types of drift-diffusion modelling. (1) In IV modelling, we apply an external voltage *V*_appl_ over the top and the bottom of the nanowire and record the current that flows through the nanowire. Furthermore, at zero external bias, we obtain the modelled built-in voltage, which can be compared to the built-in voltage extracted from electron holography. (2) In spatially-resolved internal quantum efficiency (SIQE) modelling, we keep the external bias at zero voltage, include a localized excess carrier generation source inside the nanowire, and record how many charge carriers are extracted as current [18]. We define SIQE as the ratio of extracted charge carriers to the amount of excess carriers injected by the local generation source. Then, SIQE can be compared to the measured EBIC curve, also quantitatively, assuming that the peak of the measured EBIC curve corresponds to 100% extraction of the electron-beam-induced excess charge carriers.

#### 2.5.1. Mobility

In our drift-diffusion modelling, we take into account the doping-induced modification of the charge-carrier mobility with the empirical model in Ref. [34], assuming the above *T* = 300 K. Then, we have $\mu_{e/h,n/p}=\mu_{min,e/h}+\frac{\mu_{max,e/h}-\mu_{min,e/h}}{1+{\left(N_{n/p}/N_{ref,e/h}\right)}^{\lambda_{e/h}}}$ where e/h denotes electrons/holes and n/p denotes the n or p region, with parameters from Ref. [34], as summarized in Table 1. For the n region, we use $N_{n}=N_{D}$, and for the p region, $N_{p}=N_{A}$.

#### 2.5.2. Recombination

We assume that non-radiative recombination dominates the overall recombination. This assumption is supported by the doping values that we extract below, which are low enough, implying that Auger recombination is negligible, and the non-radiative recombination parameter in our modelling which needs to be set so high that it indicates that radiative recombination is negligible. See Ref. [35] for additional details of such an assumption of non-radiative recombination dominating overall recombination in nanowires. In our modelling, we use a Shockley-Read-Hall-type non-radiative recombination mechanism with a single trap level (whose energy is assumed to coincide with the Fermi energy in an intrinsic semiconductor), with the same lifetime $\tau_{rec}$ for electrons and holes. In our 1D model, $\tau_{rec}$ can show dependence on the axial position, and both surface and bulk recombination are included through $\tau_{rec}$—see Equation (3) in Ref. [35] for details of how surface recombination enters through this recombination lifetime.

## 3. Results and Discussion

We start by presenting the characterization results in Section 3.1, Section 3.2, Section 3.3, Section 3.4 and Section 3.5. After that, in Section 3.6, we use the drift-diffusion model of the nanowire for an additional analysis of the characterization results to extract further quantitative information about the configuration of the nanowire pn-junction and recombination lifetime.

### 3.1. IV Measurements

**Figure 2:**
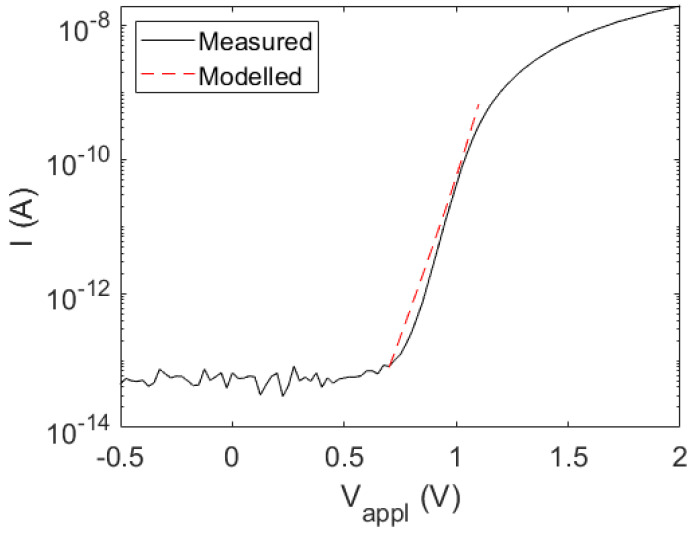
Measured (solid line) and modelled (dashed line) current vs. voltage for a nanowire (see Figure 1b for a schematic). At *V*_appl_ > 1.1 V, we see the onset of series resistance in the order of 30 MΩ. Note that there is a very minor offset in the zero value of the measured current.

The IV curve for a nanowire is shown in Figure 2. The behavior is typical of a diode. We find that this diode opens up at approximately 0.7 V, with an exponential increase in the current with increasing voltage after that, until series resistance sets in at approximately 1.1 V. Therefore, it is the 0.7 < *V*_appl_ < 1.1 V range that reveals the clearest facts about the operation of this nanowire diode.

### 3.2. Electron Holography

**Figure 3:**
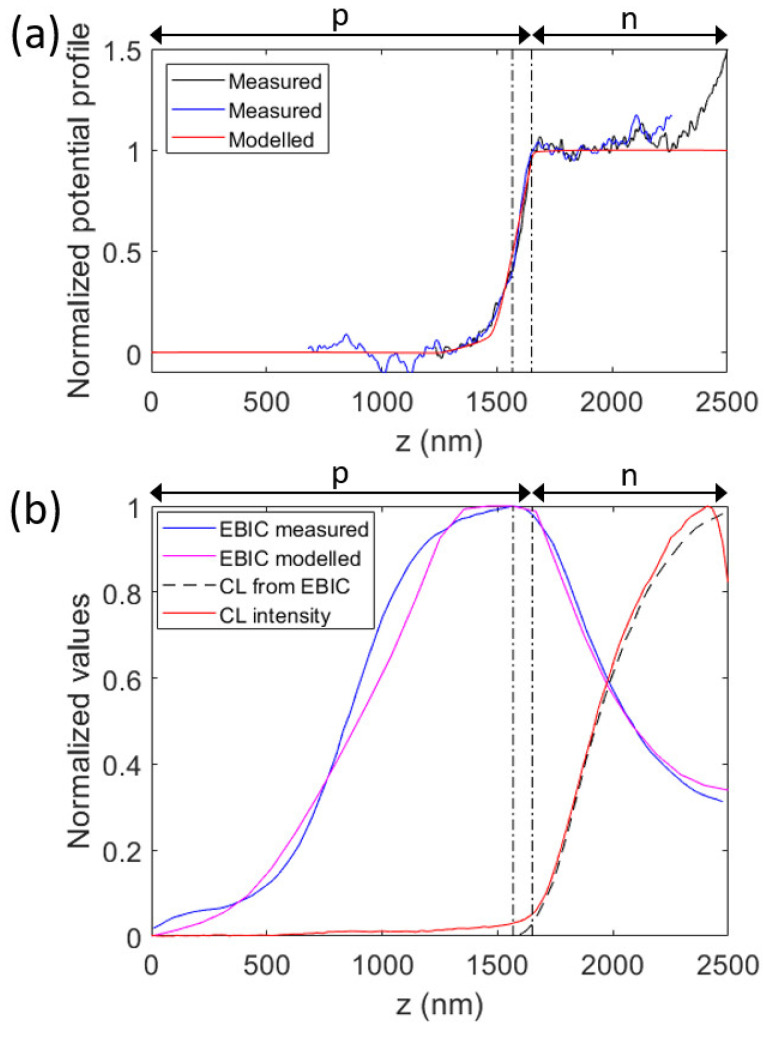
(**a**) Normalized electron potential profile extracted from electron holography as a function of axial position *z* in a nanowire. The large increase in the potential at *z* ≈ 1600 nm is assigned to the pn-junction. The second line scan, with smaller *z* values, is included to show the continuation of the profile away from the pn-junction. Here also, normalized values for the potential from our drift-diffusion model are shown, for Vappl = 0, that is, zero applied voltage. The measured spectra are shifted such that each of their mean value for *z* < 1250 nm is equal to zero, and after that they are normalized such that each of their mean values for 1700 < *z* < 2000 nm are equal to one. The modelled potential profile is shifted such that the value at *z* = 0 is at zero, before normalizing to the maximum value. (**b**) Measured and modelled EBIC and measured CL intensity as a function of the axial position in the nanowire, with *z* = 0 indicating the bottom of the nanowire at the substrate surface and $z=z_{max}=2500\;nm$ at the top of the GaAs core of the nanowire. The CL intensity is integrated for photon energies in the 1.12 to 1.85 eV range. The EBIC peaks at $z=z_{peak}=1567\;nm$. The dashed line shows values from a model that corresponds to the assumption of diffusion-dominated transport and constant recombination lifetime in the n region, with perfect FSB at the top of the nanowire (in which case a direct relation exists between the CL intensity and EBIC signal—see the main text for details). The dashed–dotted vertical lines are placed at *z* = 1567 nm and at *z* = 1650 nm to guide the eye. The arrows with p and n mark the p side and n side.

The potential profile extracted from electron holography is shown in Figure 3a. Here, the pn-junction can be seen as the rapid variation in the potential profile at *z* ≈ 1600 nm. The slope of the potential profile is proportional to the electric-drift field, which aids in the separation of charge carriers, and which is thus expected to lead to high EBIC values. Indeed, the location of the pn-junction as indicated by the potential profile coincides well with the region where the EBIC profile peaks (as discussed below in Section 3.3).

We note that the increase in the potential profile appears to start at *z* ≈ 1250 nm, which is the position according to the growth design where the dopant precursor was changed from the p type to n type. The potential profile returns to a flat profile at *z* ≈ 1650 nm (except for the increase at the very top of the nanowire, which we assign to a thin, axial AlGaAs segment, originating from the AlGaAs growth used for the radial surface passivation), indicating that the dopant concentration is constant on the n side for larger *z* values than this. In the continuation, when we refer to the n side, we refer to the region with *z* > 1650 nm.

### 3.3. EBIC Measurements

The (normalized) EBIC profile is shown in Figure 3b. The peak of the EBIC occurs at a height of approximately 1570 nm from the substrate surface. For a sharp pn-junction, with constant p and n doping around the junction and a constant lifetime, we would expect exponential decay, with the diffusion length, of the EBIC signal being away from the junction (see Section 3.2.1 in Ref. [35] for the effect of pn-junction design on modelled SIQE, which is probed using EBIC measurements). Our results indicate that our nanowire does not show such a simple, abrupt pn-junction. We believe that such a gradient in doping occurs due to the reservoir effect for dopants in the Au catalyst particle [36].

The rather high value of 0.32 for the EBIC at the top of the nanowire indicates that a front-surface barrier (FSB) is present, which prevents the electron-beam-induced excess minority carriers (that is, holes in the top n region) from reaching the top metal contact where they would recombine efficiently (which would lead to EBIC → 0 at the top of the nanowire, see Section 3.2 in Ref. [35] for details)—in other words, the FSB functions as an electron-selective contact. This FSB is probably due to the thin axial segment of the high-bandgap AlGaAs that was used for the surface passivation; effects from such an axial AlGaAs segment are visible also in the electron holography measurements, as discussed above. We expect the axial AlGaAs segment on top of the GaAs n region to be n doped due to memory effect of dopants in the Au catalyst particle during growth, in which case the conduction band of the AlGaAs and GaAs regions align, while the bandgap difference causes an efficient hole-blocking effect (see Section 3.5 in Ref. [18] for further details).

Since the EBIC profile does not decay exponentially for *z* < 1570 nm, we have indication that the p segment shows variation in the axial direction in *N*_A_ and/or $\tau_{rec}$.

### 3.4. Dependence of CL Intensity along Nanowire Axis and Comparison to EBIC Signal

The CL intensity as a function of axial position is shown in Figure 3b. On the n side, the CL signal increases as the EBIC signal decreases when moving away from the pn-junction. This behavior matches the expectation that the diffusion of excess minority carriers to the pn-junction, to which the EBIC signal is proportional, and the recombination, to which the CL signal is proportional, are competing processes (in other words, (i) a high EBIC signal leads to fewer excess minority carriers that are not extracted as an external current, and (ii) those non-extracted excess minority carriers are the ones leading to net recombination that is probed by CL. Therefore, a high EBIC signal is expected to result in a low CL signal and vice versa). If we assume a diffusion-dominated transport and constant recombination lifetime in the n region, with perfect FSB at the top of the nanowire, we can write a straight-forward quantitative relationship between the CL signal and EBIC signal. In that case, the CL signal is found using $1-\frac{EBIC\left(z\right)-EBIC(z_{max})}{EBIC\left(z_{peak}\right)-EBIC(z_{max})}$. The dashed line in Figure 3b shows values from that model for the CL signal. Indeed, we find good agreement between the measured CL intensity profile and this model.

However, on the p side, when moving away from the pn-junction, both the EBIC and CL signal drop. Thus, here, a similar simple connection between EBIC and CL intensity profiles does not show up as on the n side. Again, we thus have an indication that the configuration, in terms of recombination-lifetime profile and/or doping profile in the axial direction, of the p side is more complicated than on the n side.

### 3.5. Extraction of Doping Concentration from CL Measurements

From the CL characterization, we can extract doping concentration with a model that relates characteristics of the CL spectrum to doping concentrations. Here, the doping concentration on the respective sides of the pn-junction was estimated using the full width at half maximum (FWHM) of the bandgap-related-peak $E_{FWHM}$ from room-temperature short-circuited hyperspectral CL measurements, using $p={\left(\frac{E_{FWHM}-B}{1.68\;\cdot \;10^{-8}\;eV}\right)}^{3}\;{cm}^{-3}$ and $n={\left(\frac{E_{FWHM}-B}{3.4\;\cdot \;10^{-14}\;eV}\right)}^{\frac{3}{2}}{cm}^{-3}$ for p- and n-type doping, respectively. Refs. [37,38] describe similar estimation equations. This model is based on CL data of p-type and n-type bulk substrate reference samples of various and known concentrations. Typically, a model based on FWHM is given as a simple power law using a scaling factor, but, in our case, a broadening parameter has been used, since we aimed for a general model that would take into account the effects from the quality of surface passivation. That is, our model is intended to enable the estimation of doping concentrations for varying quality of the surface passivation. For well-passivated nanowires (such as in our case with a thick, high-bandgap AlGaAs passivation shell), the broadening parameter *B* becomes 37 meV from the fitting to reference samples (note that this value is close to the typical FWHM value for intrinsic bulk GaAs [39], and thus the model is intended only for noticeably doped samples, since for low-doped samples approaching the intrinsic linewidth, the model will provide a very low, or even unphysical, doping concentration). With poorer passivation, surface states will affect the linewidth, and the broadening term will increase. The full calibration approach goes beyond the scope of this paper and is planned for a separate publication.

Then, with the measured $E_{FWHM}$, we find on the n side a rather constant *N*_D_ ≈ 2.4 × 10^18^ cm^−3^, with a mild decrease to 1.9 × 10^18^ cm^−3^ at *z* = 1650 nm (see Figure S2 in the Supplementary Materials). On the p side, we find a rather constant *N*_A_ with the lowest value of 9.3 × 10^18^ cm^−3^ and the highest value of 1.2 × 10^19^ cm^−3^ (see Figure S2 in the Supplementary Materials). Extraction of the doping profile within the junction region from the CL FWHM is not trivial due to the depletion of free carriers and the very strong drift of excess charge carriers in that region.

### 3.6. Analysis of Characterization Results with the Help of Drift-Diffusion Model

For further analyses of the characterization results, we have built a drift-diffusion model for the nanowire. As a starting point, we will use the following characteristics extracted from the measurements above:

1. We assume a transition to the n type region at *z* = 1650 nm, with *N*_D_(*z*) having the rather constant value of 2.4 × 10^18^ cm^−3^, with exact values provided from the CL characterization (see Figure S2 in the Supplementary Materials). For this n region, we assume a constant *τ*_rec_. This assumption of a constant *τ*_rec_ is motivated by the good agreement in Section 3.2 between the CL intensity profile and the modelled values from the EBIC profile that also used the assumption of a constant *N*_D_ and *τ*_rec_ on the n side (producing the dashed line in Figure 3b).
2. We assume an FSB at the top of the nanowire, as indicated by the EBIC profile which stays at a value of >0.3 there, instead of dropping to zero as expected if no FSB was present (as discussed in Section 3.2). We include this FSB in the model through an n-doped AlGaAs segment at *z* > 2500 nm before the top contact, which is placed at *z* = 2600 nm.
3. For the p-type region, we use the *N*_A_(*z*) extracted from the CL measurements (see Figure S2 in the Supplementary Materials) for 0 < *z* < 1250 nm. This value of *z* = 1250 nm is motivated by the growth recipe described in Section 2.1 where the dopant is switched from p type to n type nominally at this value for *z*. Furthermore, the potential profile from the electron holography starts to vary from a flat profile at this *z* = 1250 nm (Figure 3a). Importantly, if we use the *N*_A_ extracted from CL also for 1250 < *z* < 1500 nm, we are unable to reproduce the electron holography and the EBIC profiles (see Figures S3 and S4 in the Supplementary Materials).

Thus, in our drift-diffusion model for the nanowire, we have as unknown parameters (i) *τ*_rec_ in the n region, and this *τ*_rec_ should be independent of *z*, (ii) values for *τ*_rec_(*z*) for *z* < 1650 nm, and (iii) values for *N*_A_(*z*) for 1250 < *z* < 1650 nm. These parameters should be tuned in such a way that we can recreate the characterization results from Section 3.1, Section 3.2, Section 3.3, Section 3.4 and Section 3.5.

We start by varying the doping profile for 1250 < *z* < 1650 nm in order to recreate the potential profile measured with electron holography in Figure 3a. Importantly, the modelled potential profile is not dependent on *τ*_rec_(*z*), since we model the potential profile at *V*_appl_ = 0. Here, it is important to note that the measured potential profile in Figure 3a provides for us a built-in voltage for the pn-junction of approximately 1.0 V (as obtained from the non-normalized measurement data underlying the normalized data shown in Figure 3a). However, such a low built-in voltage corresponds, for example, to *N*_A_ ≈ 1 × 10^15^ cm^−3^ and *N*_D_ ≈ 1 × 10^15^ cm^−3^ in GaAs, as obtained from semiconductor equations [16]. Thus, it appears that the built-in voltage extracted from the measurements in Figure 3a is noticeably lower than expected. Similar, lower-than-expected values for the built-in voltage for nanowires from electron holography have been seen also in other studies [26]. Therefore, when comparing measured and modelled potential profiles, we use normalized profiles.

For the doping gradient profile, we chose to use erf, the error function. Then, since we have from the CL measurements *N*_A_ at the beginning of the pn-junction, we have just a scaling parameter *L*_erf_ for the argument to the erf to adjust. With *N*_A_(*z*) = *N*_A_(*z*_0_)(1 − erf{[*z* − *z*_0_]/*L*_erf_)}) for 1250 < *z* < 1650 nm, with *z*_0_ = 1250 nm as the start of the graded region, we find rather good agreement between the measured and modelled potential profile with *L*_erf_ = 130 nm (Figure 3a).

Now that we have fixed the doping profile in the model, we turn to consider the remaining free parameter, that is, the possibly *z*-dependent *τ*_rec_(*z*) for *z* < 1650 nm and the constant value for *τ*_rec_ for *z* > 1650 nm.

**Figure 4:**
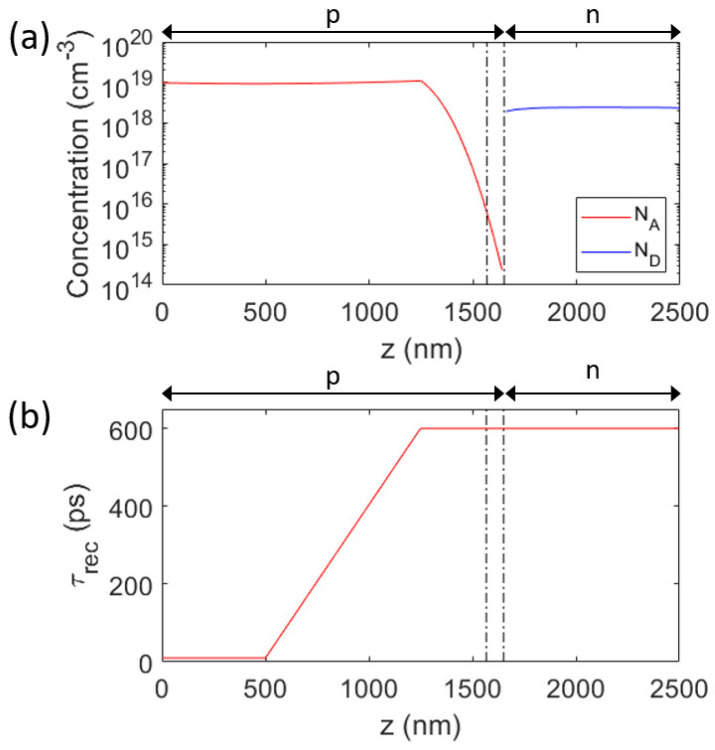
(**a**) The *N*_A_(*z*) and *N*_D_(*z*) and (**b**) the *τ*_rec_(*z*) used in the drift-diffusion model. Here, *τ*_rec_ = 10 ps for *z* < 500 nm. The dashed–dotted vertical lines are placed at *z* = 1567 nm and at *z* = 1650 nm to guide the eye. The arrows with p and n mark the p side and n side.

We proceed as follows: (i) on the n side, we find that *τ*_rec_ = 600 ps provides good agreement between measured and modelled EBIC profiles (Figure 3b). (ii) Next, we assume first a position-independent *τ*_rec_ also for the bottom part of the nanowire, with the aim of finding a value that reproduces in the modelling the measured IV curve. During this testing, we found that a value of *τ*_rec_ = 600 ps also in the pn-junction area (where the recombination in forward bias occurs), provides good agreement between measured and modelled IV curves. (iii) To allow for possible position dependence in *τ*_rec_ for *z* < 1250 nm, we used this constant *τ*_rec_ = 600 ps for *z* > 1250 nm, since the recombination in the forward direction was found to occur predominantly in the 1250 < *z* < 1650 nm region (see Figure S6 in Supplementary Materials). In this way, we allow for a possible gradient in *τ*_rec_ for z < 1250 nm without deteriorating the good agreement between the modelled and measured IV curves found in (ii). First, when we attempted to use a constant *τ*_rec_ for *z* < 1250 nm, we did not find good agreement with the measured EBIC profile (see Figure S5 in the Supplementary Materials). However, with *τ*_rec_ = 10 ps for *z* < 500 nm and a linear increase in *τ*_rec_ from 10 ps at *z* = 500 nm to 600 ps at *z* = 1250 nm, we find rather good agreement between the measured and modeled EBIC profiles also on the p side (see Figure 3b). The resulting good agreement between the measured and modelled IV curves is shown in Figure 2. The extracted values for *N*_A_(*z*), *N*_D_(*z*), and *τ*_rec_(*z*) are shown in Figure 4.

## 4. Conclusions

By combining the IV, EBIC, CL, and electron holography measurements with the drift-diffusion model of the nanowire, we could reveal a plausible configuration for the nanowire (Figure 4). From CL measurements, we obtained estimates for *N*_A_(*z*) for *z* < 1250 nm and for *N*_D_(*z*) for *z* > 1650 nm. By varying *N*_A_(*z*) for 1250 < *z* < 1650 nm with the aim to recreate the electron potential profile measured with electron holography, we ended up with the concentration profiles shown in Figure 4a. From EBIC and CL measurements on the n side, we had a strong indication that *τ*_rec_ is rather constant there. With drift-diffusion modelling, we found good agreement between the measured and modelled EBIC with *τ*_rec_ = 600 ps in the modelling in the n region. The IV curve of the nanowire diode is predominantly dependent on *τ*_rec_ in the pn-junction. This fact allowed us to vary *τ*_rec_ in the 1250 < *z* < 1650 nm junction area, without noticeable influence of the *τ*_rec_ set for *z* < 1250 nm. We found that the same *τ*_rec_ = 600 ps as was used for the n region, when used in this junction region, provided good agreement between the measured and modelled IV curves (Figure 2). Lastly, by aiming to recreate the measured EBIC profile in the p segment for *z* < 1250 nm through modelling, we found good agreement when using a.graded *τ*_rec_ (Figure 4b).

However, we must point out that the drift-diffusion model that we developed above for the nanowire does not appear to be absolutely perfect. With that model, we were not able to reproduce the CL intensity profile on the p side (see Figure S7). We leave it as an open question to future studies to investigate this discrepancy. Could the discrepancy for example originate from the effects of radial band-bending due to Fermi-level pinning at the surface of the GaAs nanowire core [40]? Such radial effects cannot be easily included in the 1D model that we used for the nanowire and would need dedicated additional experiments to allow extension of the model to one with both axial and radial dependence.

In conclusion, this work opens the possibility to recreate the internal structure of a nanowire from the information obtained from one or several characterization techniques. The number and type of characterization techniques that are needed are expected to depend on the complexity of the nanowire. Here, we demonstrated how to use modelling aid to simulate data for the analysis of, e.g., CL, EBIC, IV, and electron holography characterization results, with additional input from the known, nominal nanowire growth design. Such an analysis framework is expected to streamline material and device development in nanowire-based applications.

## Figures and Tables

**Table 1:** Mobility parameters for GaAs from Ref. [34].

Quantity	Value
$N_{ref,e}$	$6.0\times 10^{16}\;{cm}^{-3}$
$N_{ref,h}$	$1.48\times 10^{17}\;{cm}^{-3}$
$\lambda_{e}$	0.394
$\lambda_{h}$	0.38
$\mu_{min,e}$	$500\;{cm}^{2}/Vs$
$\mu_{min,h}$	$20\;{cm}^{2}/Vs$
$\mu_{max,e}$	$9400\;{cm}^{2}/Vs$
$\mu_{max,h}$	$491.5\;{cm}^{2}/Vs$

## Data Availability

All the necessary data are included in the article.

## Supplementary Materials

The following supporting information can be downloaded at https://www.mdpi.com/article/10.3390/mi15010157/s1, Figure S1: Measured CL spectra. Figure S2: Doping concentration from CL; Figure S3: Additional modelling of potential profile; Figure S4: Additional modelling of EBIC profile; Figure S5: Additional modelling of EBIC profile; Figure S6: Spatially resolved recombination rate; Figure S7: Modelled CL intensity. Table S1. Summary of the steps and key parameters for the nanowire growth.
