# Supplementary material for: Analysis of Nanowire pn-Junction with Combined Current–Voltage, Electron-Beam-Induced Current, Cathodoluminescence, and Electron Holography Characterization
Source: Micromachines (Basel). 2024 Jan 20;15(1):157. doi: 10.3390/mi15010157 (PMC10819615; doi:10.3390/mi15010157)
Supplement: Supplementary file 1 [file micromachines-15-00157-s001.zip › micromachines-2772628-supplementary.pdf]

Supplementary Materials: Analysis of Nanowire pn-Junction with Combined Current–Voltage, Electron-Beam-Induced Current, Cathodoluminescence, and Electron Holography Characterization

Nicklas Anttu [1], Elisabetta Maria Fiordaliso [2], José Cano Garcia [3],

Giuliano Vescovi [3], and David Lindgren [3]

[1]Physics, Faculty of Science and Engineering, Åbo Akademi University, FI-20500 Turku, Finland;

[2] Center for Electron Nanoscopy, Technical University of Denmark, Denmark

[3] Sol Voltaics AB, Lund, Sweden

Email: nicklas.anttu@abo.fi

S1. Nanowire Growth

Core-shell nanowire arrays were grown by the vapor-liquid solid (VLS) method on p-type GaAs (111)B substrates in a low pressure metal-organic vapor phase epitaxy (MOVPE) system with a total flow of 13 l/min using hydrogen (H2) as carrier gas. An array of circular Au discs forming a square pattern of Au catalyst particles with a pitch of 500nm was defined on the surface by nano imprint lithography (NIL). SiN_x growth mask was included for pattern preservation during growth.

The reactor was first heated up to 375 °C under arsine mixture (AsH_3/H_2) with arsine molar fraction of 7.69×10^{-4}. Then trimethylgallium (TMGa) was introduced to the reactor for 90 s for prebake nucleation of GaAs. Subsequently, the temperature was raised to 650 °C for a 5 min annealing under AsH_3/H_2 mixture with arsine molar fraction of 1.15×10^{-3}, after which the temperature was lowered to 400 °C and nanowire growth started with a 60 s p-GaAs nucleation under the flow of AsH_3, TMGa and DEZn with molar fractions of 7.69×10^{-4}, 1.03×10^{-4}, and 5.74×10^{-7} respectively. After p-GaAs nucleation, a 15 s Zn depletion step consisted of switching off diethylzinc (DEZn) flow and ramping AsH_3 flow to molar fraction of 9.43×10^{-3} in an attempt to deplete the Au particle of Zn. Subsequently, i-GaAs growth was continued at these molar fractions of AsH_3 and TMGa for another 260 s. Following i-GaAs, an n-GaAs segment was grown for 45 s by reducing AsH_3 and TMGa flows to molar fractions of 2.00×10^{-3} and 2.23×10^{-5}, and introducing the flow of triethyltin (TESn) with molar fraction of 1.07×10^{-5}. The core growth was finally completed by additional 140s of i-GaAs growth with AsH_3 and TMGa molar fractions as in the first i-GaAs segment growth.

After the core GaAs nanowire growth, reactor temperature and pressure were raised to 715 °C and 400 mbar, as a preparation for the shell growth. AsH_3 flow was increased to a molar fraction of 1.38×10^{-2}. The nanowires were then annealed for 5 min at 715 °C under the AsH_3/H_2 flow mixture. The AlGaAs radial shell was then grown for 120 s by introducing TMGa and trimethylaluminum (TMAl) flows with molar fractions of 4.37×10^{-5} and 1.57×10^{-4}. Finally, to protect the AlGaAs shell from oxidation, a thin GaAs cap was grown for 120 s by switching off TMAl. The growth was ended by cooling down under AsH_3 flow with molar fraction of 3.85×10^{-3}. See Table S1 summarizing the growth steps and the key parameters.

From calibration runs, we have indication that the memory effect [1] of Zn in the Au particle causes p doping throughout the first i-segment. Similarly, the second i-segment appears n doped. Thus, the growth recipe in Table S1 is intended to create a nanowire with constant p doping in the bottom part of the nanowire and constant n doping in the top part of the nanowire, with sharp transition from n to p doping.

Table S1. Summary of the steps and key parameters for the nanowire growth.

	T (C)	AsH_3	TMGa	DEZn	TESn	TMAl	P (mbar)	Time (s)
Heating up under AsH_3	375	7.69E-04					100	until T==375
Prebake nucleation	375	7.69E-04	2.67E-04				100	90
Ramp up T		1.15E-03					100	
Bake	650	1.15E-03					100	300
Ramp down T		1.15E-03					100	
pGaAs nucl	400	7.69E-04	1.03E-04	5.74E-07			100	60
Deplete Au of Zn	400	7.69E-04-> 9.43E-03	1.03E-04				100	15
i-GaAs	400	9.43E-03	1.03E-04				100	260
n-GaAs	400	2.00E-03	2.23E-05		1.07E-05		100	45
i-GaAs	400	9.43E-03	1.03E-04				100	140
Ramp up T, p		9.43E-03 -> 1.38E-02					100-> 400	
anneal	715	1.38E-02					400	300
AlGaAs shell	715	1.38E-02	4.37E-05			1.57E-04	400	120
GaAs cap	715	1.38E-02	4.37E-05				400	120
Cool down		3.85E-03					400	

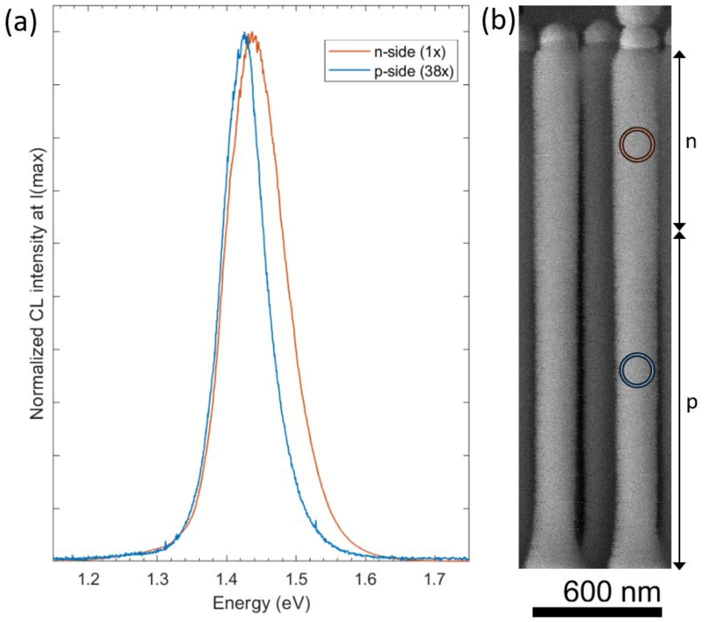

Figure S1. (a) CL intensity, as a function of photon energy, at approximately the middle of the n-side and p-side. Here, the peak intensity on the n-side is 38 times higher than on the p-side. (b) SEM image with the circles indicating the position of the CL measurement in (a) for the n-side (top circle) and p-side (bottom circle). The arrows with p and n mark the p-side and n-side.

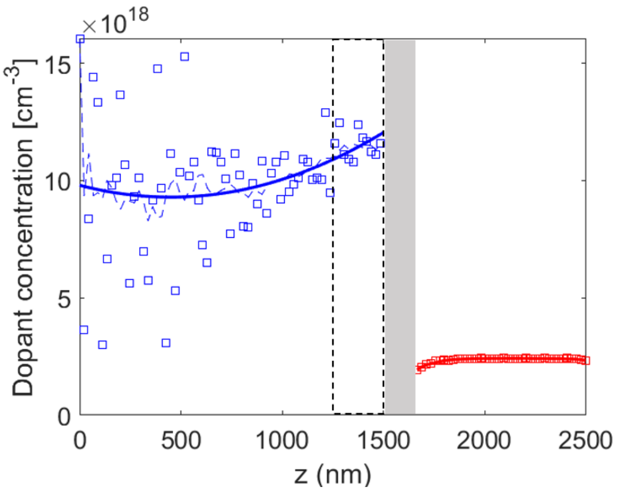

Figure S2. Doping concentration extracted from CL. On the n-side (red squares) we find a rather constant $N_D \approx 2.4\times10^{18}$ cm^{-3} except for a slight decline to 1.9×10^{18} cm^{-3} at z = 1650 nm. The solid red line shows a moving average, which we use as input to the drift-diffusion model. On the p-side (blue squares), we find close to the pn-junction $N_A \approx 1.2\times10^{19}$ cm^{-3} with a mild variation toward the bottom of the nanowire, with lowest value of $N_A \approx 9.3\times10^{18}$ cm^{-3}. The dashed blue line shows a moving average over 10 adjacent measurement points. The solid blue line is a second order polynomial fit, which we use in the drift diffusion model in the main text. The greyed-out region indicates the pn-junction region where free carrier density is depleted and where a strong drift field exists (see Figure 3a). The dashed region marks the $1250 < z < 1500$ nm region where electron holography and EBIC indicate a drift field, as discussed in the main text.

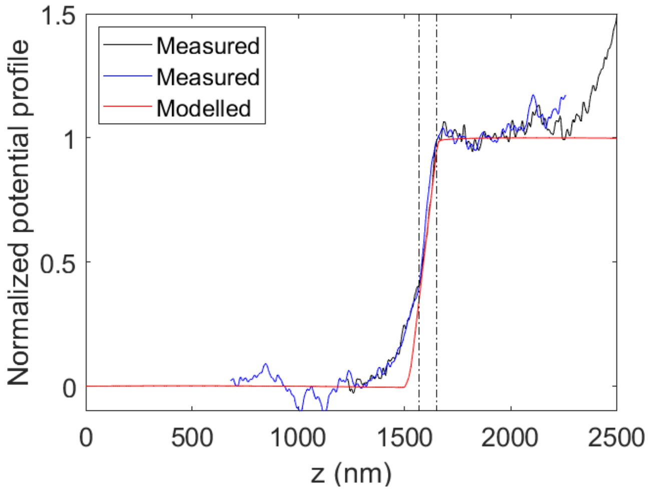

Figure S3. Normalized electron potential profile extracted from electron holography as a function of axial position z in a nanowire, as well as normalized values from our drift-diffusion model, here for $V_{appl} = 0$, that is, zero applied voltage. The measured spectra are shifted such that each of their mean value for z < 1250 nm is zero, and after that they are normalized such that their mean value for 1700 < z < 2000 nm is equal to one. The modelled potential profile is shifted such that the value at $z = 0$ is at zero, and then normalization is performed to the maximum value. Here, the values for $N_A(z)$ for $z < 1500$ nm from Figure S2 were used in the model. We used in the model $N_A(z) = N_A(z_0)(1 - \text{erf}\{[z - z_0]/L_{erf}\})$ for $1500 < z < 1650$ nm, with z_0 = 1500 nm as the start of the graded region and L_{erf} = 20 nm. Similarly as in Figure 3a, the dashed-dotted vertical lines are placed at z = 1567 nm and at z = 1650 nm to guide the eye.

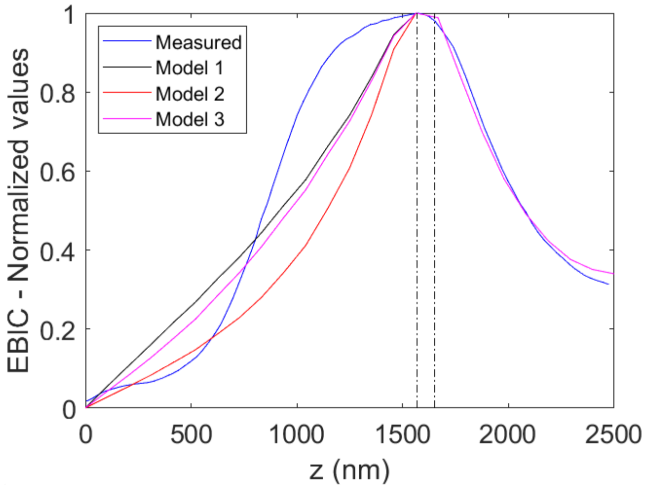

Figure S4. Measured and modelled EBIC. In contrast to the model in the main text, in this modelling, we use the N_A distribution that gives the modelled potential profile in Figure S3. In further contrast to the model in the main text, in Model 1 we use constant τ_{rec} = 600 ps for z < 1500 nm, in Model 2 τ_{rec} = 100 ps for z < 1500 nm, and in Model 3 τ_{rec} = 100 ps for z < 500 nm, τ_{rec} = 600 ps for 1000 < z < 1500 nm, and for 500 < z < 1000 nm, we use linear variation from 100 ps to 600 ps. For all three models, the maximum modelled EBIC value is 1, within numerical uncertainty. Similarly as in Figure 3, the dashed-dotted vertical lines are placed at z = 1567 nm and at z = 1650 nm to guide the eye.

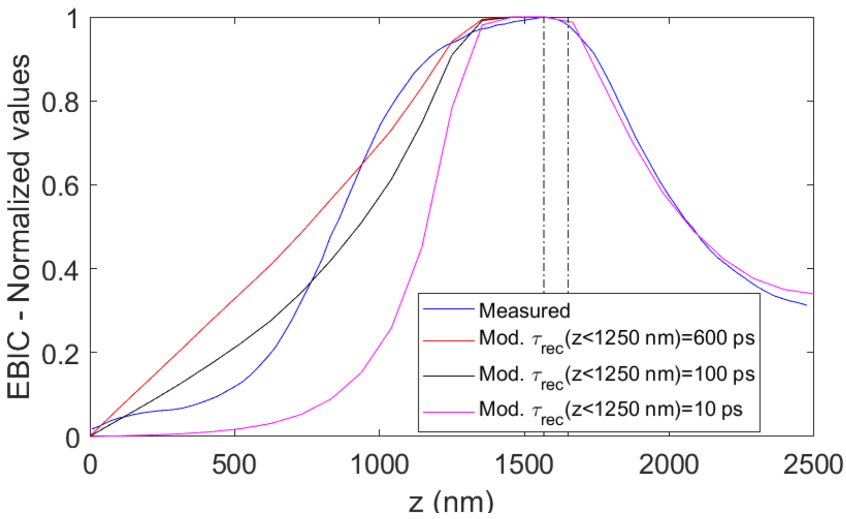

Figure S5. Measured and modelled EBIC. In contrast to the model in the main text, in the three modelled curves, we use constant τ_{rec} = 600 ps, τ_{rec} = 100 ps, and τ_{rec} = 10 ps, respectively, for z < 1250 nm. For all three models, the maximum modelled EBIC value is 1, within numerical uncertainty. Similarly as in Figure 3, the dashed-dotted vertical lines are placed at z = 1567 nm and at z = 1650 nm to guide the eye.

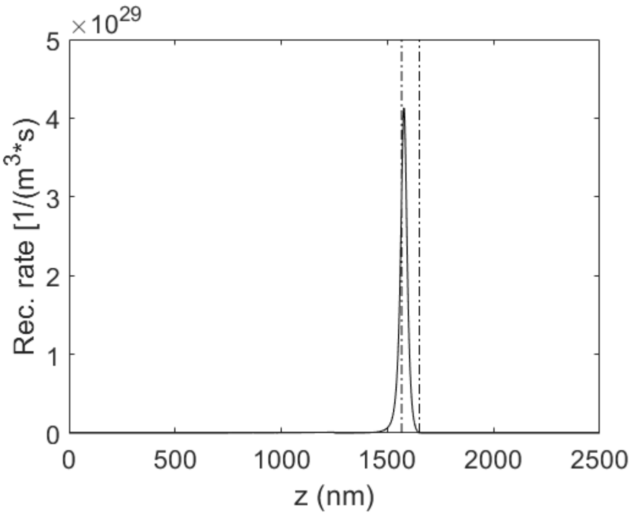

Figure S6. Spatially resolved recombination rate from the drift-diffusion model at V_{appl} = 1.0 V. Similarly as in Figure 3, the dashed-dotted vertical lines are placed at *z* = 1567 nm and at *z* = 1650 nm to guide the eye.

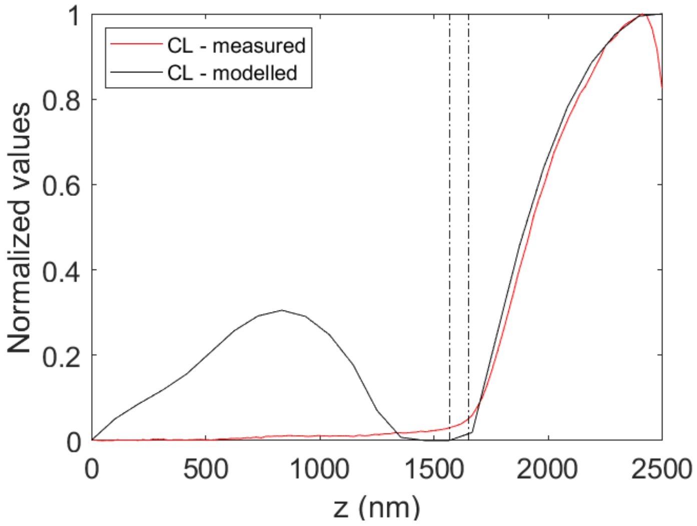

Figure S7. Measured CL intensity together with modelled CL intensity from the drift-diffusion model for the nanowire (see Figure 4). In the model, we assume for simplicity that the CL intensity is given by the expression for radiative recombination in a non-degenerate semiconductor: $R_{\mathrm{rad}} = \int_0^{2500\ \mathrm{nm}} B\,(n(z)p(z) - {n_{\mathrm{i}}}^2)\mathrm{d}z$, with n the electron concentration, p the hole concentration, and n_{i} the intrinsic carrier concentration [2]. Since we consider normalized values, the value of the radiative recombination constant B does not enter the analysis explicitly. To model the CL intensity at varying *z* position, we induce additional photogeneration rate at that position, similarly as in the EBIC modelling—this excess photogeneration rate induces a variation to $n(z)$ and $p(z)$ from the equilibrium values, giving rise to a non-zero value for R_{rad}. Similarly as in Figure 3, the dashed-dotted vertical lines are placed at *z* = 1567 nm and at *z* = 1650 nm to guide the eye.

References

1. Kim, W.; Güniat, L.; Fontcuberta i Morral, A.; Piazza, V. Doping Challenges and Pathways to Industrial Scalability of III–V Nanowire Arrays. *Appl. Phys. Rev.* **2021**, *8*, 011304.
2. Sze, S.M.; Li, Y.; Ng, K.K. *Physics of Semiconductor Devices*; John Wiley & Sons: Hoboken, NJ, USA, 2021; ISBN 978-1-119-61800-3.
